# Co-existence of thin basement membrane nephropathy with other glomerular pathologies; a single center experience

**DOI:** 10.12860/jnp.2015.09

**Published:** 2015-04-01

**Authors:** Rizwan A. Qazi, Bahar Bastani

**Affiliations:** ^1^Department of Internal Medicine, Division of Nephrology, University School of Medicine, Saint Louis, USA

**Keywords:** Familial hematuria, Glomerulonephritis, Hematuria, IgA nephropathy, Thin basement membrane nephropathy

## Abstract

*Background:* The co-existence of thin basement membrane nephropathy (TBMN) and another glomerular pathology portends a worse prognosis than TBMN alone.

*Objectives:* The purpose of our study was to investigate the prevalence of TBMN and associated glomerular pathologies at our institution.

*Patients and Methods:* We reviewed all renal biopsies performed at Saint Louis University hospital over a 7-year period. We excluded all post transplant biopsies, and biopsies showing diabetic glomerulopathy, membranoproliferative glomerulopathy, membranous glomerulopathy, and biopsies where no electron microscopy or immunofluorescent studies were done. All other biopsies were included.

*Results:* A total of 634 biopsies were included in the study. The prevalence of TBMN was 47 (7.4%), of whom 17 (36.2%) had TBMN alone. In the remaining 30 (63.8%) patients TBMN was associated with other glomerular pathologies: IgAN 9 (19.1%) and FSGS 9 (19.1%). We found significantly higher prevalence of IgAN in patients with TBMN versus all biopsies (19.1% vs. 7.7%, respectively, *P* = 0.002). We found significant similarities in biopsy indications for TBMN and IgAN group.

*Conclusions:* Around two thirds of the cases of TBMN were associated with other glomerular pathologies. The prevalence of IgAN, but not focal segmental glomerulosclerosis, was significantly higher in patients with TBMN as compared to the general renal biopsy specimens.

**Implication for health policy/practice/research/medical education:**

While glomerular thin basement membrane disease alone has a benign course, the cases that come to the attention of nephrologists, because of proteinuria or renal dysfunction, are often complicated with other glomerular pathologies that would affect the course of the disease.

## 1. Background

Thin basement membrane nephropathy (TBMN), also known as familial benign hematuria, is the leading cause of persistent microscopic hematuria in children and adults. It is due to a mutation in collagen 4 – alfa 3 or 4 genes (1,2). Persistent hematuria is thought to be due to passage of red blood cells through the transient gaps in the abnormally thin glomerular basement membrane. Proteinuria is

usually not present because of the increased absorptive capacity of proximal tubular cells in these patients. Other clinical features of TBMN are hypertension, which is quite common ranging between 11% to 31% (3-6), and renal insufficiency that is not very common since pure TBMN is not considered to be a progressive disease. When TBMN occurs with heavy proteinuria, renal insufficiency and hypertension, a co-existing glomerular pathology is usually present (5-9). The co-existence of TBMN and another glomerular pathology portends a worse prognosis as compared to TBMN alone. However, there is no evidence that TBMN alters the prognosis of a concomitant glomerulopathy (9).

Patients with IgA nephropathy (IgAN) have clinical presentation similar to patients with TBMN with most patients presenting with painless hematuria and variable degrees of proteinuria. Most nephrologists will not biopsy a patient with pure hematuria and no proteinuria; however, the threshold for doing a kidney biopsy is reduced when patients have significant proteinuria. TBMN and IgAN are both very common glomerular diseases in the general population. If a patient has hematuria with mild proteinuria he/she is more likely to have either TBMN or IgAN (9,10).

## 2. Objectives

Since the first diagnosed case of TBMN in 1974, there have been a number of case reports, case series and data base queries, trying to link the pathogenesis of TBMN with other glomerular pathologies based on the fact that some glomerular pathologies (e.g. IgAN, FSGS) seem to occur more commonly than others in this group of patients (6-12). The purpose of our study was to investigate if such an association existed at our institution.

## 3. Patients and Methods

### 3.1. Data collection

We reviewed the reports of all kidney biopsies performed between January 1999 and January 2006. Patients’ age, sex, indications for renal biopsy, and review of the pathological diagnosis were done with particular attention to the details of light microscopy, immunofluorescence, and electron microscopy. All adults (> 18 years of age) biopsies were included. Any biopsy where electron microscopy or immunofluorescence staining were not done was excluded. All transplant biopsies and biopsies that had revealed diabetic glomerulopathy, membranous (MGN) or membranoproliferative GN (MPGN) were also excluded. A total of 634 biopsies were included in our study. In our institution, it is a standard practice to do special stains for different type IV collagen-alfa chains in patients who are found to have TBMN. This is done to rule out Alport’s syndrome.

Thin basement membrane was defined using standard World Health Organization (WHO) criteria, which requires diffuse thinning (<250 nm) of the glomerular basement membrane in adults (13).

### 3.2. Ethical issues

The research was approved by the Saint Louis University Institutional Review Board.

### 3.3. Statistical analysis

The difference in prevalence of IgAN in TBMN group vs. general group was calculated using chi-square. SAS was used to evaluate the differences in biopsy indications in TBMN and IgAN. A *P*-value of <0.05 was considered statistically significant.

## 4. Results

After the above-mentioned exclusions we had 634 renal biopsies with their related clinical information. 315 (49.7%) were males and 319 (50.3%) were females. The prevalence of different renal pathologies was as follows: TBMN 47 (7.4%), IgA nephropathy 49 (7.7%), FSGS 118 (18.6%), focal global glomerulosclerosis (FGGS) 7 (1.1%), mesangioproliferative GN 12 (1.9%). The remaining 401 (63.3%) had other pathologies (e.g., lupus nephritis, interstitial nephritis, acute tubular necrosis, hypertensive nephrosclerosis, etc).

Fifty-three patients were found to have thinning of the glomerular basement membrane, 5 of which had focal thinning, and 1 patient had Alport’s syndrome. These 6 patients were excluded, and thus we were left with 47 patients who met the criteria for TBMN according to WHO criteria. In this group of patients there were 37 (78.7%) females and 10 (21.3%) males. Average (±SD) age was 43.7 ±16 years. Of the 47 cases with TBMN, 17 (36.2%) had TBMN alone; in the remaining 30 (63.8%) cases, TBMN was associated with other glomerular pathologies.

The prevalence of different glomerular pathologies associated with TBMN was as follows: IgAN 9 (19.1%), FSGS 9 (19.1%), mesangioproliferative GN 2 (4.3%), lupus nephritis 1 (2.1%), pauci-immune crescentic GN 3 (6.4%), acute interstitial nephritis 2 (4.2%), focal endocapillary proliferative glomerulonephritis 1 (2.1%), acute endocapillary glomerulonephritis 1 (2.1%), chronic sclerosing GN 1 (2.1%), and one patient had TBMN with IgA and FSGS. We found significantly higher prevalence of IgAN in patients with TBMN versus all biopsies (19.1% vs. 7.7%, respectively, *P* = 0.002). The prevalence of FSGS in the TBMN group was 19.1% that was not significantly different from its prevalence (18.6%) in all biopsies. In the 118 patients with FSGS, 9 (7.6%) patients had TBMN that was similar to the 7.4% prevalence of TBMN in all biopsies.

Indications for kidney biopsy (all biopsies, those with TBMN, or IgAN) are shown in  Table 1 . Hematuria alone was indication of biopsy in 22.4 % of IgAN, 21.3% of TBMN, and only 4.1% of all biopsies. This difference was highly significant (*P* < 0.005) when TBMN and IgAN groups were individually compared with all biopsies. Other statistically significant differences in the indications for biopsy among the three groups (all biopsies vs TBMN or IgAN) were: Hematuria and proteinuria 19.8% vs. 32%, 32.7%, respectively (*P* < 0.01), and renal failure 15.9% vs. 4.2%, 4.1%, respectively (*P* < 0.005), Table 1 . Although both groups (TBMN & IgAN) were significantly different from all biopsies with regards to indications for biopsy (i.e., hematuria, hematuria & proteinuria, and renal failure), the two groups were very similar to each other in terms of indications for biopsy. Four patients in the TBMN group had nephrotic range proteinuria (3 with co-existing FSGS, one with collapsing FSGS). Interestingly, none of the 39 patients, among all biopsies, who had history of diabetes mellitus had any evidence of diabetic glomerulopathy on biopsy, however 7 (18%) of these 39 showed IgAN on biopsy.

**Table 1:** The indications for renal biopsy in all patients (general), TBMN and IgAN

	**Indications**	**General n=634**	**TBMN n=47**	**IgAN n=49**
1.	Hematuria alone	26 (4.1%)	10 (21.3%)^a^	11 (22.4%) ^a^
2.	Hematuria + proteinuria	126 (19.8%)	15 (32.0%) ^b^	16 (32.7%) ^b^
3.	Hematuria + renal failure	20 (3.2%)	4 (8.5%)	3 (6.1%)
4.	Proteinuria alone	172 (27.1%)	12 (25.5%)	6 (12.2%)
5.	Proteinuria + renal failure	167 (26.3%)	4 (8.5%)	11 (22.4%)
6.	Renal failure alone	101 (15.9%)	2 (4.2%) ^a^	2 (4.1%) ^a^

^a^ *P* < 0.005 TBMN & IgAN vs. general; b *P* < 0.01 TBMN & IgAN vs. general.

## 5. Discussion

In this study we report 47 cases of TBMN with a prevalence rate of 7.4% among 634 biopsies performed over a 7-year period at our center. This is consistent with the previously reported 5% to 10% prevalence rate of TBMN in general population and in the donor specimens undergoing “time zero” biopsies (7,14). Previously published data show increased prevalence of certain glomerular pathologies like IgAN or FSGS in this subgroup of patients (7,9,10). We also found a significantly higher prevalence of IgAN, but not FSGS, in our TBMN patients. Moreover, we compared the indications for biopsy in all 634 biopsies versus the TBMN and IgAN sub-groups. We found very similar clinical indications for biopsy between the TBMN and IgAN versus all biopsies (Table 1). The association of IgAN with TBMN has been noted in a number of cases reports, case series and data base queries (7,9,10,12,15,16). It has been called a syndrome by some authors, and thought to have a pathogenic link by others (9,12).

Patients with TBMN and another glomerular pathology usually have more pronounced proteinuria, hematuria, hypertension and renal insufficiency when compared to those who have pure TBMN (5-9,17,18). TBMN was previously called benign familial hematuria because of its relatively benign nature. However, only one third of our patients had pure TBMN, and hence a “benign” and non-progressive nature, and the other two third had some associated glomerular pathology, which could worsen their prognosis. The high prevalence of other glomerular pathologies in the present series and the previous reports of TBMN is due to a selection bias in favor of biopsying patients with more significant renal involvement than a mild isolated microscopic hematuria. Moreover, a recent clinicopathological report on 127 patients from 11 large pedigrees found a definite association of heterozygous COL4A3/COL4A4 mutations with familial microscopic hematuria, chronic renal failure and end stage renal disease, due to FSGS, suggesting that the term “benign familial hematuria” is a misnomer, at least in their cohort (19,20).

IgA nephropathy is considered to be due to a qualitative defect in IgA molecule, i.e., defective glycosylation at the hinge region of the IgA1 molecule. In one study the investigators showed that the abnormal glycosylation of the IgA molecule that occurs in IgA nephropathy is absent in patients with combined TBMN and IgA nephropathy (21). These investigators suggested that the mechanism of IgA deposition in the mesangium might differ in patients with TBMN and in patients with classic IgA nephropathy. This may point to a pathogenic association of IgA with TBMN.

Focal or segmental thinning of GBM is common in patients with acquired glomerulopathies (22-24). This has been attributed to an injury-repair mechanism. In a series of 26 patients with TBMN, segmental TBMN accounted for one third of their series (24). Focal-global glomerulosclerosis was more common in diffuse TBMN. It was proposed to define TBMN as a clinicopathological entity of dysmorphic hematuria and a diffusely or segmentaly thinned GBM confirmed by the direct measurement technique (24).

In our case series the prevalence of FSGS in the sub-group of patients with TBMN was not different from that in the general biopsy specimens; and vice versa, the prevalence of TBMN in biopsies with FSGS was not different from that in general biopsy specimen. Moreover, we had excluded patients with focal thinning of glomerular basement membrane since focal thinning per se does not constitute TBMN.

Our study has the limitations inherent of any retrospective chart review. We also excluded patients with certain glomerular pathologies (i.e., MGN, MPGN, DM) and those who did not have electron microscopy or immunofluorescence studies. Some of these patients could have had underlying thin basement membrane disease.

## 6. Conclusions

We have shown an increase prevalence of IgAN in patients with TBMN. Whether IgAN in the presence of TBMN is a secondary phenomenon or has a pathogenic connection remains to be elucidated.

## Acknowledgements

We acknowledge the most valuable contribution of late Professor Luis Salinas-Madrigal from the Department of Pathology at Saint Louis University School of Medicine in reviewing all pathologic specimens in the present study.

## Authors’ contributions

Collecting data: RAQ, BB. Statistical analysis: RAQ, BB. Drafting manuscript: RAQ, BB. Study supervision: BB.

## Conflict of interests

The author declare no conflict of interest.

## Funding/Support

None.
